# Membrane Hormone Receptors and Their Signaling Pathways as Targets for Endocrine Disruptors

**DOI:** 10.3390/jox12020007

**Published:** 2022-03-25

**Authors:** Yves Combarnous, Thi Mong Diep Nguyen

**Affiliations:** 1 INRAe, CNRS, Tours University Joint Unit, Physiologie de la Reproduction et des Comportements, 37380 Nouzilly, France; nguyenthimongdiep@qnu.edu.vn; 2 Faculty of Natural Sciences, Quy Nhon University, Quy Nhon 820000, Vietnam

**Keywords:** tyrosine kinase receptor, serine/threonine kinase receptor, cytokine receptor, G protein-coupled receptor, guanyl cyclase receptor, ion-channel receptor, orthosteric site, allosteric site, estradiol, bisphenols

## Abstract

The endocrine disruptors are mostly small organic molecules developed for numerous and very diverse industrial applications. They essentially act through nuclear receptors with small and hydrophobic endogenous ligands. Nevertheless, potential adverse effects through membrane hormone receptors cannot be ruled out, and have indeed been observed. The present paper reviews how orthosteric and allosteric binding sites of the different families of membrane receptors can be targets for man-made hydrophobic molecules (components of plastics, paints, flame retardants, herbicides, pesticides, etc.). We also review potential target proteins for such small hydrophobic molecules downstream of membrane receptors at the level of their intracellular signaling pathways. From the currently available information, although endocrine disruptors primarily affect nuclear receptors’ signaling, membrane receptors for hormones, cytokines, neuro-mediators, and growth factors can be affected as well and deserve attention.

## 1. Introduction

More than 85,000 chemicals are registered in commerce, and recent estimates have identified approximately 1000 of them as potential endocrine disruptors (EDs) [1]. Most well-recognized EDs are small organic molecules impacting cell signaling through nuclear receptors (NR) [2]. For example, the estrogen receptors (ER), androgen receptor (AR), thyroid hormone receptors (TR), aryl hydrocarbon receptor (AhR), peroxisome proliferator-activated receptors (PPARs), and many others are physiologically specific for small hydrophobic endogenous mediators. The EDs exhibit structures resembling those of small-size hormones or other mediators, and can thus act as agonists or antagonists, either by direct binding with these NRs or indirectly by interfering with the binding of their endogenous cognate mediators with blood transport proteins, cell importers, or enzymes involved in their synthesis or degradation [3].

In contrast to the NRs, the membrane receptors (MbR) generally accommodate hydrophilic ligands of very variable sizes, from about 100 in MW such as glycine to more than 500,000, such as adiponectin polymers. Can they also be targets of endocrine disruptors?

Membrane receptors belong to various structural families with either only one transmembrane domain (TyrK-R, Ser/ThrK-R, cytokine family receptors, guanyl cyclase receptors, etc.), or several (ion-channel receptors, 7TM-R including G protein-coupled receptors (GPCR), TNFR, etc.). These receptors exhibit different structures and intracellular signaling pathways that can both be impacted differently by endocrine disruptors.

Compared to NRs, which are transcription factors [4], MbRs exert acute short-term responses even when they ultimately stimulate gene transcription. Considering the classical definitions of EDs [5,6,7,8], there is no theoretical impeachment for MbRs to be ED targets. Nevertheless, there is only limited literature dealing with such cases. 

The potential EDs targeting membrane receptors are expected to interact either at their orthosteric site (the specific site for their endogenous ligand) or at remote allosteric site(s), inducing change(s) in the receptor conformation [9] or stabilizing either its active or inactive conformation [10]. Orthosteric sites are located at the surface of cell plasma membranes to allow access to their circulating endogenous ligands. In contrast, most allosteric sites are located in the transmembrane domains, which convey information from outside towards inside the cells through conformational changes.

Positive allosteric modulators (PAMs) bound to allosteric sites potentiate the activity of the agonist bound at the orthosteric site. Negative allosteric modulators (NAMs) inhibit the agonist activity in the same way. In contrast, the silent allosteric modulators (SAMs) exert no effect on the activity of the agonist bound at the orthosteric site but can inhibit the binding of PAMs or NAMs [11]. 

Allosteric site predictions generally focus on communication signals propagating from the allosteric sites to the orthosteric sites. However, recent biochemical studies have revealed that allosteric coupling is bidirectional and that orthosteric perturbations can modulate allosteric sites through reversed allosteric communication [12]. In the frame of the present paper, it means that certain molecules could bind to receptors’ cryptic sites that would become accessible only in the presence of the cognate hormone at the orthosteric site [13]. Such molecules would therefore not be EDs by themselves but only in the presence of the endogenous stimulating hormone.

Pharmaceutical companies search druggable receptor sites to develop molecules that can interfere, positively or negatively, with the signaling of hormones or other mediators [14]. Most current drugs bind directly to the orthosteric sites of their target proteins (mainly receptors), whereas less used allosteric modulators can elicit a wider variety of biological responses. All these sites, either ortho- or allo-steric, can also be considered potential binding sites for disruptors.

By definition, the membrane receptors are inserted in direct interaction with the plasma membrane bilayer lipids. Modulating lipid composition is a potential way for EDs to exert a disturbing effect on membrane receptor receptivity and/or activity [15,16].

In the present short review, we focus on demonstrated and putative mechanisms of action of already known and potential EDs interfering directly at the level of membrane receptors or downstream of ligand binding. Among potential EDs, we include the pharmaceuticals targeting membrane receptors’ allosteric sites in case they would (themselves or their metabolites) become present at a significant concentration in the environment.

## 2. Tyr-Kinase Receptors (RTKs)

Trans-non-alachlor, chlordane, DDE, DDT, dieldrin, alachlor, atrazine, lindane, PCB-153, and PCB-126 have been found to interact with the typical RTK epidermal growth factor receptor (EGFR) [17] and, consequently, to inhibit constitutive androstane receptor (CAR) activity [18], leading to adverse outcomes in sex organs and metabolic organs. Indeed, CAR is a ligand-activated regulator of xenobiotic-metabolizing enzymes, and its inhibition synergizes the effects of most EDs. In particular, its EGFR-mediated inhibition synergizes the effects of most endocrine disruptors and other xenobiotics.

Insulin resistance is a key feature of Type 2 Diabetes (T2D), and improving insulin sensitivity is important for disease management. Therefore, an exciting challenge is the allosteric modulation of the insulin receptor (IR) with drugs [19,20]. As put forward before, drugs can lead to EDs emergence by dispersion of the drugs or their active metabolites in the environment. Small molecules have been designed to target the juxta-membrane domain of RTKs, which activates signaling by these receptors. For instance, gambogic amide binds to the intracellular juxta-membrane region of the nerve growth factor receptor TrkA, thereby inducing receptor dimerization and activation [21]. As allosteric pathways are conserved among RTKs [22], the discovery of similar drugs for the other family members can be expected, together with the possible risk of appearance of new EDs.

Endocrine disruptors can also affect the RTKs’ downstream signaling pathways. For example, the phenylsulfamide fungicide tolylfluanid has been shown to induce cellular insulin resistance in primary murine and human adipocytes by reducing insulin receptor substrate-1 (IRS-1) expression and stability [23].

## 3. Ser/Thr-Kinase Receptors (STKRs)

For the time being, very few examples of allosteric modulation of STKR structure have been described [24]. Therefore, to our knowledge, no drug targeting these receptors has been commercialized so far. Nevertheless, small organic inhibitors (lY-2157299, SB-431542, and a-83-01) of some type 1 activin receptors (ALK) are being developed to inhibit tumor and stem cells’ multiplication [25], indicating that organic molecules could potentially become EDs through this pathway.

Downstream of a STKR, the carbamate pesticide carbofuran inhibits neuron stem cell proliferation and neuronal differentiation by stimulating the TGF-β signaling pathway (Smad-2, -3, and -7 and Smurf-2) in the rat hippocampus [26]. Additionally, a recent study established the molecular mechanism of arsenical toxicity at the level of Smad2/3 activity [27], which is the classical downstream signaling pathway of this class of receptors. Acute exposure to arsenite leads to cytosolic retention of activated Smad2/3 essentially due to augmented Smad2/3 nuclear exportation relative to its import. This leads to dampening of the TGF-β downstream signaling.

## 4. Cytokine and Related Receptors

The receptors for cytokines (interleukins, toll-like, tumor necrosis factor, etc.) or for the hormones GH, PRL, EPO, Leptin, etc., possess only one transmembrane domain and require dimerization for activation when their orthosteric site is occupied. Upon dimerization or polymerization, they, directly (ILRs, GHR, PRLR, EPOR) or indirectly (TLRs, TNFR), recruit and activate cytoplasmic protein kinases such as Jak, Tyk, Lck, IRAK, etc., and, for some of them (TNFR), caspase cascades.

The extracellular domain of the human prolactin receptor (hPRL-R) uses an identical epitope to bind to both prolactin (hPRL) and growth hormone (hGH). Very subtle structural changes in the extracellular domain of hPRL-R induced by the binding of each hormone at the same orthosteric site are sufficient to determine the biological output triggered by each hormone-binding [28]. 

As many as 32 druggable sites were found by the cosolvent mapping method in 25 representative conformations of the IL-1R1 cytokine receptor [14]. These druggable sites are also potential binding sites for disruptors. 

There is some evidence that “classical EDCs” (phthalates, bisphenol-A, octyl- and nonyl-phenol, vinclozolin, atrazine, etc.) are especially problematic in the peri-conception phase of pregnancy when the maternal immune response is first established, and the critical events of implantation and early placentation occur [29]. In many cases, these EDs act through the nuclear estrogen receptors present in immune cells [30]. Nevertheless, possible effects through various specific immune receptors cannot be excluded. Indeed, cholesterol is a negative allosteric regulator of the TcR, as it only binds to TcRs in their resting conformation, thus stabilizing the resting state [31]. It can be expected that hydrophobic molecules competing with cholesterol at this site would affect TcR function in immune cells. Moreover, BPA has a profound effect in disturbing the immune responses of macrophages by attenuating the release of pro-inflammatory cytokines and inflammatory mediators by the MAPK and NF-κB signaling pathways downstream of TcR [32]. 

Concerning TNF signaling, large-scale networks of TNFR trimers must form from pre-ligand assembled dimers for stimulation. Small organic molecules can bias the ensemble of TNFR1 conformational states toward those states associated with activation [33,34,35]. A small-size activator of TNFR1 signaling, named SB 200646 hydrochloride (SBH), has been identified and indicates that small hydrophobic molecules should be able to, intentionally or not, play a similar role.

## 5. Seven-Transmembrane Domain Receptors (7TM-R/GPCR)

The human genome encodes roughly 350 GPCRs, and around half of all modern medicinal drugs target them. Indeed, GPCRs are prominent targets for pharmaceuticals acting either at their orthosteric or allosteric sites [36]. The existence of allosteric ligands has enriched how the functions of GPCRs can be manipulated with potential new drugs.

These drugs or their metabolites can become EDs when present in the environment and unintentionally ingested. 

In GPCR, the ligand orthosteric sites can be located inside the transmembrane domain (deep-pocket) or at the cell surface at the level of the loops between successive transmembrane peptides or formed by an N-terminal extracellular domain (ECD). Depending on the orthosteric site location, the allosteric sites can be present at any other location in the receptor structure.

Interestingly, GPCRs can be targeted by “classical EDs” (those already shown to act via NRs), in particular in the hypothalamo-pituitary gonadal axis of vertebrates [37]. In addition, since GPCRs are numerous not only in the endocrine system but also in the nervous and immune system, endocrine disruptors can potentially provoke some neurological and immunological defects through GPCRs in addition to nuclear receptors.

The glycoprotein hormone receptor TSHR has been found to possess a putative allosteric site accommodating compounds such as DDT, DES, or quercetin [38]. Various compounds are currently developed to target allosteric sites in glycoprotein hormone receptors, particularly the FSHR [39,40]. Agonists such as thiazolidinones, diketopiperazines, hexahydroquinolines, and thienopyrimidines, and antagonists such as sulfonic acid, (bis)sulfonic acid, (bis)benzamides, tetrahydroquinolines, and benzamide derivatives, are being tested [41]. Indeed, glycoprotein hormones are highly complex molecules. They are challenging to produce and handle, and the search for simpler activating molecules is very active. As pointed out before, GPCRs, like all membrane receptors, can be sensitive to their lipid environment. 

The GPCR Ghrelin/GHSR-1a/growth hormone secretagogue receptor (GHSR) exhibits sensitivity to PIP2 [15], which is also the substrate for phospholipase C (PIP2 > DAG + IP3) and for PI3-kinase (PIP2 > PIP3). This phosphoinositide (PIP2)b plays a central role in several signaling pathways, and its direct interaction with GPCRs can physiologically be essential. This phosphoinositide stabilizes the active conformation of GHSR, and foreign hydrophobic molecules that would interfere with PIP2 concentration in membranes or with its various effects would disturb numerous cellular signaling pathways. In particular, allosteric modulators may change receptors’ active conformation and lead towards biased signaling [39]. This bias would be due to changes in the relative efficacies of their downstream signaling pathways.

Among the 7TMRs, the membrane estrogen receptor (mbER, GPR30, GPER) is suspected as an additional, prominent target for the classical EDs [42] such as bisphenol A (BPA) and many others [43], acting essentially through the nuclear ERs. Low doses of BPA promote human seminoma cell proliferation by activating PKA and PKG also via the membrane GPER [44]. In invertebrates [45] and vertebrates [46,47], BPA impairs the development of the serotonin-5-HT-immunoreactive brain or intestinal neurons and can be considered as neuro-disruptors in this context. Many EDs also exert metabolism-disturbing activity as a consequence of their ED activity or because of their toxicity during early development [1].

The adipokin receptors are 7-TM receptors but not GPCRs. In contrast to the large majority of 7-TM receptors, their C-terminus is extracellular whereas their N-terminus is cytoplasmic. Certainly, this explains that they do not interact with trimeric G-proteins (Gs, Gi, Gq, etc.). Instead, they stimulate the AMPK pathway through CAMKKβ activation [48]. AMPK is a central regulator of cellular energy homeostasis and is the target for numerous small molecules [49] intended to combat several cancer types and diabetes. Among them, metformin has emerged as a largely prescribed drug and has become of concern as an aquatic pollutant ED in fish [50]. 

GPCRs operate through various G protein-dependent signaling pathways, among which adenylate cyclase activation through Gs (>cAMP) and phospholipase C activation through Gq (>DAG + IP3 > Ca++) are the most well-studied. The cAMP-dependent kinase PK-A phosphorylates the CREB transcription factor, which is central to many hormone-controlled effects and which has been shown to be rapidly activated by low doses of BPA [51]. Likewise, BPA has also been shown to affect the phosphoinositide pathway downstream of IP3 by direct interaction with the endoplasmic reticulum-located IP3 receptor [52]. The herbicide atrazine has been shown to inhibit cAMP-phosphodiesterase-4 in pituitary and Leydig cells and thus to act as an ED through a MbR pathway [53]. 

## 6. Guanyl Cyclase Receptors

The cardiac hormone atrial natriuretic peptide (ANP) possesses a receptor in the kidney, which is the best-known receptor with guanylate cyclase activity. ANP increases the glomerular filtration rate, antagonizes the renin-angiotensin-aldosterone system, and hinders sodium reabsorption at the level of the renal collecting duct. ANP has been identified recently as being associated with chronic kidney disease (CKD) without diabetes through the stimulation of secretion of cytokines (IL-6, TNF-α) and adiponectin [54]. ANP is also strongly expressed in several cancers (in the skin, ovary, prostate, and stomach) [55], but has also been described to possess antitumor activity [56]. These properties indicate that any impact, positive or negative, on ANP signaling by pollutants, including EDs, might increase the number of cancer cases. 

In addition, epidemiological studies have attributed approximately 40% of the population burden of hypertension to obesity, and low ANP levels in such individuals have been invoked as a potential mechanism [57]. Despite its involvement in all these critical pathologies, no effective drug, except one currently in development [44], has targeted ANP receptors. In agreement with this lack of drugs, no EDs targeting guanylate cyclase receptors have been identified.

## 7. Ion-Channel Receptors

The ion-channel receptors can be classified by their gating and the nature of ions passing through their channel. Examples of such channels include the cation-permeable “nicotinic” acetylcholine receptor (nAChR), ionotropic glutamate-gated receptors, acid-sensing ion channels (ASICs), ATP-gated P2X receptors, and the anion-permeable γ-aminobutyric acid-gated GABA_A_ receptor. Most of these receptors are located in synapses between neurons or at neuro-muscular or neuro-glandular junctions. For example, if such receptors are located in the thalamus, hypothalamus, pineal gland, or adrenals, disruptors acting at their level might behave as endocrine disruptors.

Ligand-gated ion-channel receptors can potentially be affected by molecules structurally resembling their endogenous ligands, but they can also be affected by molecules interacting with other sites. The nAChR has been nicknamed “typical allosteric machine” [58], and molecules such as Ca++ or the anthelmintic ivermectin and anesthetics can bind to other sites and positively or negatively affect the response to ACh. Disruptors can thus potentially act on ion-channel receptors through allosteric sites not only in the nicotinic nAChR [59] but also in the transient receptor potential (TRP) ion channels [60] and many others. 

The CMPI compound (3-(2-chlorophenyl)-5(5-methyl-1-(piperidin-4-yl)-1H-pyrrazol-4-yl)isoxazole) binds to a non-canonical nAChR binding site at the β2:β2 interface and not at the canonical site at the α4:β2 interface. CMPI has only very weak stimulating activity by itself but strongly potentiates stimulation by ACh and nicotine [61]. 

TRP channels are a superfamily of ion channels that are sensitive to diverse chemical and physical stimuli and play diverse roles in biology (there are 27 *trp* genes in humans). The compound 2-APB (2-aminoethoxydiphenyl borate) modulates the activity of many of them through a common allosteric mechanism outside their orthosteric site [60]. Additionally, the antimicrobial triclosan has been shown to interact with the TRPA1 channel and stimulate VEGF secretion in human prostate cancer cells [62]. 

Moreover, some ion channels can also be directly affected by steroid hormones [63] and therefore by molecules resembling them, such as numerous classical EDs.

In brief, ion-channel receptors, with allosteric site(s) at their transmembrane domains, could accommodate various hydrophobic molecules of industrial origin, able to modify their response properties.

## 8. Non-Receptor Membrane Proteins

Transforming growth factor-beta receptor III (TGFBR3), also known as beta-glycan, is a cell-surface chondroitin sulfate/heparan sulfate proteoglycan of >300 kDa in molecular weight that can bind TGF-β with low affinity and high capacity. It is not an actual receptor as it does not directly convey intracellular signaling. It is merely a reservoir or kind of a sponge attracting TGF-β at the surface of target cells, close to TGF-β-RI and -RII, which are the actual receptors upstream of the Smad signaling pathway. Since most industrial pollutants are hydrophobic, the probability is low that they can interact with the highly hydrophilic polysaccharide chains of beta-glycan. It is nevertheless not excluded that these molecules can interact with the transmembrane protein part of beta-glycan.

A large number of classical EDs have been shown to affect membrane proteins involved in cell–cell adhesion and communication (cadherins, catenins, claudins, connexins, etc.) [64], but the mechanisms involved (direct interaction and/or effect on their expression) are not yet clear.

## 9. Discussion

The overview presented in this short review clearly indicates that “classical” endocrine disruptors might express their deleterious effects through membrane receptors, although most of them do it essentially through nuclear receptors [2]. Membrane hormone receptors (MbRs) are much more diverse than nuclear receptors and their ligands are also very different in size and physico-chemical properties (charge, hydrophobicity, etc.).

**Figure 1:**
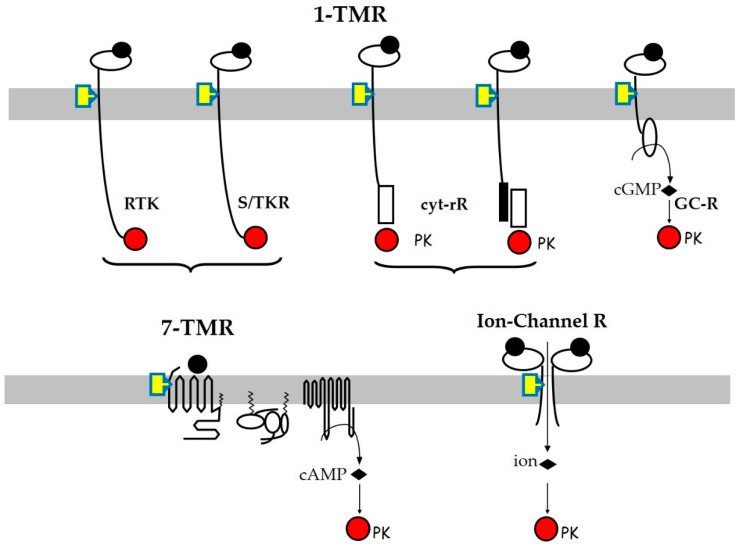
Schematic view of membrane receptor structures and their signaling pathways. The target cell plasma membranes are shown in grey, the finally impacted protein kinases (PK) in red, and the ligands as black dots occupying the receptor orthosteric sites. Most endocrine disruptors, which are hydrophobic molecules, mainly act through allosteric sites located in the transmembrane or juxta-membrane region(s) of the receptors, shown as yellow rectangles.

Figure 1 summarizes the different types of MbRs and their respective downstream signaling pathways.

Due to their anchoring in the lipid bilayer, the MbRs are generally more accessible to hydrophobic molecules at the level of their transmembrane domain(s) than at their extracellular solvent-exposed domain, where their specific ligand site (orthosteric) is present. A few MbRs are specific for hydrophobic ligands (estradiol, prostaglandins, etc.) and thus their orthosteric site might be a target for small organic molecules. More generally, MbRs are specific for large hydrophilic molecules (proteins, glycoproteins) and their orthosteric sites are not expected to be targets for hydrophobic molecules. Nevertheless, a number of MbRs have been shown to be activated or inhibited by hydrophobic molecules with structures not related to their cognate ligand. These molecules bind to receptor allosteric sites at juxta-membrane or membrane locations. They promote either stimulation of the MbRs by themselves or, more often, synergize or inhibit the endogenous hormone activity.
